# 3D pancreatic carcinoma spheroids induce a matrix-rich, chemoresistant phenotype offering a better model for drug testing

**DOI:** 10.1186/1471-2407-13-95

**Published:** 2013-02-27

**Authors:** Paola Longati, Xiaohui Jia, Johannes Eimer, Annika Wagman, Michael-Robin Witt, Stefan Rehnmark, Caroline Verbeke, Rune Toftgård, Matthias Löhr, Rainer L Heuchel

**Affiliations:** 1 CLINTEC, Karolinska Institutet, Stockholm 14186, Sweden; 2 Center of Biosciences, Karolinska Institutet, Stockholm 14186, Sweden; 3 Axcentua Pharmaceuticals AB, Nobels Allé 10, Stockholm, 14157, Sweden; 4 Department of Pathology, Karolinska Institutet, Stockholm, 14186, Sweden

## Abstract

**Background:**

Pancreatic ductal adenocarcinoma (PDAC) is the fourth most common cause of cancer related death. It is lethal in nearly all patients, due to an almost complete chemoresistance. Most if not all drugs that pass preclinical tests successfully, fail miserably in the patient. This raises the question whether traditional 2D cell culture is the correct tool for drug screening. The objective of this study is to develop a simple, high-throughput 3D model of human PDAC cell lines, and to explore mechanisms underlying the transition from 2D to 3D that might be responsible for chemoresistance.

**Methods:**

Several established human PDAC and a KPC mouse cell lines were tested, whereby Panc-1 was studied in more detail. 3D spheroid formation was facilitated with methylcellulose. Spheroids were studied morphologically, electron microscopically and by qRT-PCR for selected matrix genes, related factors and miRNA. Metabolic studies were performed, and a panel of novel drugs was tested against gemcitabine.

**Results:**

Comparing 3D to 2D cell culture, matrix proteins were significantly increased as were lumican, SNED1, DARP32, and miR-146a. Cell metabolism in 3D was shifted towards glycolysis. All drugs tested were less effective in 3D, except for allicin, MT100 and AX, which demonstrated effect.

**Conclusions:**

We developed a high-throughput 3D cell culture drug screening system for pancreatic cancer, which displays a strongly increased chemoresistance. Features associated to the 3D cell model are increased expression of matrix proteins and miRNA as well as stromal markers such as PPP1R1B and SNED1. This is supporting the concept of cell adhesion mediated drug resistance.

## Background

Over the past decades pancreatic ductal adenocarcinoma (PDAC) has become the subject of increased research activity, however, the prognosis of this disease remains the worst amongst solid tumours. The 5-year survival rate is still below 5%, and this is at least partially due to an almost complete resistance against both conventional and targeted chemotherapy. With the present standard of care, conventional chemotherapy results in a median life expectancy of around 6 months [1]. Recent evidence suggests that the molecular basis for this chemoresistance is multifaceted and reflects a wide range of genetic changes in a multitude of cellular pathways and response [2], including drug transportation [3] and microenvironmental alterations [4]. A better understanding of the underlying mechanisms is key to the identification of novel therapeutic strategies capable of overcoming this chemoresistance.

Three-dimensional culture of tumour cells was introduced as early as the 1970s. Initially, investigations focused on the morphology of and interactions between tumour cells [5]. Various PDAC cell lines were tested for their ability to grow as spheroids in 3D culture [6,7]. Among these, the widely used Panc-1, which carries both KRAS and p53 mutations, was shown to form aggregates under appropriate culture conditions [6]. It became apparent that 3D cultures are generally more resistant to chemo- and radiotherapy than their 2D counterparts [8,9], however validated three-dimensional *in vitro* tumour cell models allowing for fast and standardized drug screening are not routinely employed. Based on these observations, a new hypothesis relating chemoresistance to the microenvironment, i.e. the stroma and extracellular matrix, was proposed. This novel concept, coined *cell adhesion mediated drug resistance* (CAM-DR), was proposed for bone-marrow derived malignancies [10], but has not been applied to solid tumours, including PDAC [11]. In this study, we characterize a 3D tumour model in which the PDAC acquires a more stroma-rich phenotype, which simulates more closely the *in vivo* situation, and provides evidence for the CAM-DR concept.

## Methods

### Cell culture

The following well-characterized human pancreatic ductal adenocarcinoma cell lines (ATCC) were used: AsPC-1, BxPC-3, Capan-1, Panc-1 [6,12]. A human immortalized pancreatic stellate cell (PSC) line [13] was used as a non-transformed control cell line. KPC cells were established from a mouse PDAC model, carrying pancreas-specific Kras and p53 mutations (Kras^LSL-G12D/+^;Trp53^LSL-R172H/+^;p48-Cre; hence KPC) [14]. Cells were cultured under standard culture conditions (5% CO_2_, at 37°C) in DMEM/F12 or phenol red-free DMEM/F12 medium (Gibco) containing 10% fetal calf serum (FCS, Invitrogen).

### 3D culture

Cells were trypsin-treated and counted using the Casy Cell Counter according to the manufacturer’s recommendations (Schärfe System GmbH, Reutlingen, Germany). Subsequently, they were seeded onto round bottom non-tissue culture treated 96 well-plates (Falcon, BD NJ, USA) at a concentration of 2500 cells/well in 100 μl DMEM-F12 or phenol red-free DMEM-F12 medium, containing 10% FCS and supplemented with 20% methyl cellulose stock solution. For preparation of methylcellulose stock solution we autoclaved 6 grams of methylcellulose powder (M0512, Sigma-Aldrich) in a 500 ml flask containing a magnetic stirrer (the methylcellulose powder is resistant to this procedure). The autoclaved methylcellulose was dissolved in preheated 250 ml basal medium (60°C) for 20 min (using the magnetic stirrer). Thereafter, 250 ml medium (room temperature) containing double amount of FCS (20%) was added to a final volume of 500 ml and the whole solution mixed overnight at 4°C. The final stock solution was aliquoted and cleared by centrifugation (5000 g, 2 h, room temperature). Only the clear highly viscous supernatant was used for the spheroid assay (about 90-95% of the stock solution). For spheroid generation we used 20% of the stock solution and 80% culture medium. corresponding to final 0.24% methylcellulose. Spheroids were grown under standard culture conditions (5% C O_2_, at 37°C) and harvested at different time points for RNA isolation or drug testing as stated below.

### mRNA isolation and RT-PCR analysis

Cells or spheroids were collected, washed once with cold PBS, and processed for total RNA isolation using the RNeasy or the miRNeasy Mini Kit (Qiagen). RNA integrity and concentration were analyzed using agarose gel electrophoresis and Nanodrop Spectrophotometer. One μg of total RNA was retrotranscribed (First Strand cDNASynthesis kit, Roche). In the case of microRNA analysis, the NCode™ VILO™ miRNA cDNA Synthesis Kit (Invitrogen) was used for retrotranscription.

SYBR-Green Technology (Fermentas) was used for all qRT-PCR experiments. Further detailed information regarding qPCR reactions and oligonucleotide primers sequences is included in Additional file 1: S1.

### SDS-PAGE and western blotting

Whole cell lysates from 2D or 3D cultured cells were prepared using M-PER^®^ Mammalian Protein Extraction Reagent lysis buffer (Pierce Biotechnology, Thermo Scientific, Rockford, USA). The protein concentrations were measured using a BCA Protein Assay kit (Pierce). Cell lysates (50 μg) were resolved on 8% SDS-PAGE and analysed by immunoblotting. Anti-E-cadherin antibody was from BD transduction laboratories (BD610182, dilution 1: 2500). Anti-HIF1α antibody was from NOVUS Biologicals (NB100-449, dilution 1:500. Anti-Glut-1 and Anti-GAPDH (used as loading control) antibodies were from Abcam, Cambridge, UK (ab40084, dilution 1:2000 and ab9483, dilution 1:5000, respectively). Primary antibodies were detected with peroxidase-conjugated donkey Anti-rabbit immunoglobulin antibody (Amersham) and visualized with Immun-Star WesternC Chemiluminescence Kit (BIO-RAD) by a cooled CCD camera system (molecular Imager Chemo DocTM XRS System, BIO-RAD).

### Immunofluorescence and electron microscopy

Spheroids were harvested at fixed time points and washed twice with PBS. For immunohistochemistry, spheroids were fixed in 4% paraformaldehyde, embedded in paraffin and sectioned. Seven μm sections were stained as described below. Prior to blocking (PBS-tween 1% BSA), 0.01 M Sodium Citrate Buffer, pH 6.0, was used as an antigen retrieval solution. Anti-collagen I (rabbit polyclonal, ab292, Abcam, dilution 1:500) and Anti-fibronectin (mouse monoclonal, ab6328, Abcam, dilution 1:200) were used as primary antibodies. Biotinylated Anti-rabbit or Anti-mouse secondary antibodies from Vector Laboratories (Bulingame, CA, USA) were used in combination with streptavidin-coupled DyLight 549 from Jackson ImmunoResearch for fluorescence detection.

For electron microscopy, spheroids were fixed in phosphate buffer pH 7.4 containing 4% glutaraldehyde and 1% paraformaldehyde, and subsequently embedded and processed. Imaging was performed on a Tecnai 12 Spirit Bio TWIN transmission electron microscope (Fei Company, Eindhoven, The Netherlands) at the Central Electron Microscopy Unit of Karolinska Institutet.

### Lactate accumulation measurement

Cells were grown both in 2D and 3D culture (2500 cells/well in 96 well plates) without medium change for the whole experiment time (from day 1 to day 10). Lactate accumulation was measured in the medium of four different wells at each time point using the YSI 2700 SELECT™ Biochemistry Analyzer (YSI life sciences, Yellow Springs, Ohio, USA) according to manufacturer’s recommendations. Cell-free medium was used as a control. Mean concentrations of lactate were calculated after subtracting lactate levels measured in the cell-free medium. Cells in corresponding wells (2D or 3D cultures) were lysed with M-PER^®^ Mammalian Protein Extraction Reagent (#78501, Pierce). Protein quantification was performed using Pierce BCA protein Assay Reagent kit (#23225) and quantified with the ELISA reader (Molecular Devices Spectra MAX 250). The number of lactate moles per well was calculated from the measured lactate molar concentration, normalized for the total protein content of the cells/spheroid from the same well. The metabolite concentration was then expressed as mol/g total protein.

### Drug test, acidic phosphatase (APH) assay

For 2D culture, cells were seeded on flat bottom 96 well plates (Costar) at a concentration of 2500 cells/well in 100 μl phenol red-free RPMI-F12 medium containing 10% FCS. For 3D culture, cells were seeded according to the description for spheroid preparation in phenol-free medium. On day 4 drugs (see Table 1 and Additional file 2: S2) were added at the indicated final concentrations in an extra volume of 10 μl/well and in 8 replicates for each time point. On day 7, a slightly modified acidic phosphatase (APH) assay (see Additional file 3: S3) was performed [15]. The viability rate was calculated as a percentage of the untreated cells. All data were expressed as the mean ± SD of at least 8 replicates. All experiments were performed at least three times. To confirm the reliability of the APH assay on 3D culture, a re-growth assay was performed. After drug treatment, half of the spheroids (control and treated; 4 for each sample) were disaggregated by trypsin without chelators for fifteen minutes at 37 degrees and re-seeded as single cell suspensions on flat bottom 96 well plates for conventional 2D culture. After one day, APH assay was performed on both the 3D and the derived 2D cultures. Comparison of results demonstrated the same reduction in cell viability (data not shown).

**Table 1:** Experimental drugs used in 2D and 3D cultures with respective viabilities

**Name**	**Class**	**Concentration**	**Viability after treatment,%**	
			**2D**	**3D**
Gemcitabine	nucleoside analogue	1 μM	63	83
H107	microtubulin inhibitor	10 μM	10	97
CB5	microtubulin inhibitor	10 μM	58	93
CB7	microtubulin inhibitor	10 μM	55	100
CB13	microtubulin inhibitor	10 μM	70	95
AXP-107-11	genistein derivative	100 μM	40	65
6-MP	mercaptopurine	200 μM	53	97
6-MPR	mercaptopurine	200 μM	52	95
MT100	allicin derivative	200 μM	19	37
Allicin	diallyl thiosulfinate	200 μM	19	46
act16412	sHH inhibitor	20 μM	72	100
GANT61	sHH inhibitor	20 μM	85	100

The concentrations with the greatest effect are recorded. For details see Figure 7.

## Results and discussion

### Formation of compact 3D spheroids

**Figure 1:**
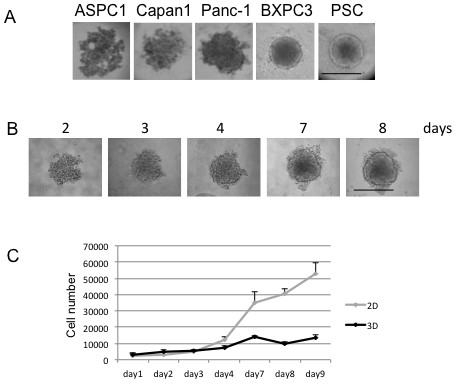
**Spheroid development. **[**A**] Different PDAC cell lines grown in 3D culture for 4 days. According to the grading system by Sipos et al.[6], Panc-1, BXPC3 and ASPC-1 are poorly differentiated and carry both mutant KRAS and p53 (Panc-1) or either p53 (BXPC3) or KRAS (ASPC-1), whereas Capan-1 is a well differentiated PDAC cell line. A previously established pancreatic stellate cell line (PSC) is also included as a non-transformed control cell line. [**B**] Development of a single representative Panc-1 spheroid, photographed from day 2 to day 8 by counting with the Boyden chamber the cell number of trypsinized spheroids and taking pictures of spheroids at fixed time points. [**C**] Cell counts from 2D and 3D cultures at different time points. Bars correspond to 500 μm.

To date, many approaches and techniques have been described for culturing cells in 3D [16]. In this study, we grew cells in the absence of exogenous ECM components, and instead, the crowding agent methylcellulose, a cellulose-derived inert compound which helps cells to aggregate and form spheroids, was added [17]. The cells built up a 3D microenvironment that closely resembles the in vivo situation (Figure 1), while avoiding the known bias that exogenous ECM components may have on cell signaling [16]. We tested various starting cell numbers per well (data not shown) and 2500 cells were found to be optimal for a 7-day growth period. This allows for sufficient ECM production and keeps the diameter below the critical size of 500 μm, when necrosis starts to develop in the spheroid center [18]. This size was in the range of what had been described regarding viability of other cancer type cells in spheroid [19]. Various PDAC cell lines were tested for their ability to form spheroids. We investigated Panc-1, MiaPaCa2, BXPC3 and ASPC-1, which are poorly differentiated [6] and carry both KRAS and p53 (Panc-1 and MiaPaCa2) or either p53 (BXPC3) or KRAS (ASPC-1) mutations. In addition, Capan-1 was included in the study as a well-differentiated PDAC cell line, and a pancreatic stellate cell (PSC) line was used as a non-transformed control cell line [13]. Of those, Panc-1 cells formed relatively compact and round spheroids whereas BXPC3 and PSC formed extremely compact spheroids with a well-defined contour (Figure 1A). In contrast, MiaPaCa2 lacked any degree of cell aggregation and ASPC-1 or Capan-1 cells were aggregating without generating a compact spheroid (Figure 1A). As the Panc-1 cell line is reported as less differentiated and more aggressive than others [6], it was selected for further testing.

The growth kinetic of Panc-1 spheroid formation was assessed longitudinally (Figure 1B). Loose cell clustering occurred on day 2, and was followed by a gradually more compact growth, until on day 4, a spheroid with a diameter of 450–500 μm had developed and remained relatively stable until day 8. Cell viability, evaluated by trypan blue staining, was approximately 90% in both 2D and 3D cultures (data not shown). The increase in cell numbers over time indicated that proliferation was reduced in 3D compared to conventional 2D culture, especially after day 4 (Figure 1C).

**Figure 2:**
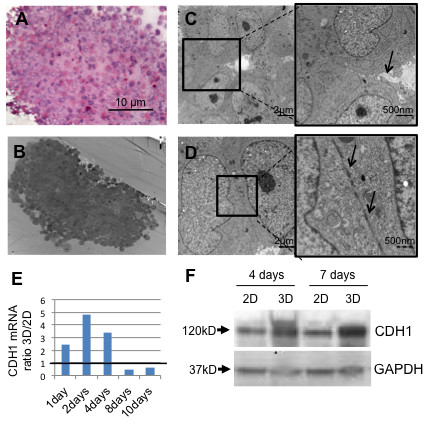
**Morphological analysis of 3D cultured Panc-1 cells. **[**A**] Hematoxilin Eosin staining and [**B-D**] electron microscopy analysis of a central 7 days spheroid section. C and D show details of the same section at EM. In C the arrow indicates the presence of a lumen. In D tight junction structures are indicated by arrows. [**E**] E-cadherin expression in both 2D and 3D culture assessed by RT-PCR. Data are calculated as expression ratio 3D/2D. [**F**] Western blotting shows E-cadherin protein expression in 2D and 3D culture on day 4 and 7.

To assess the cellular morphology, spheroids were sectioned and examined by light and electron microscopy (EM). On H&E staining cells within the spheroid sections were found to be homogeneously distributed, and, in accordance with the viability data, no or only small necrotic areas were detected (Figure 2A). Similar observations were made on EM examination (Figure 2B), which also revealed cellular arrangement around an empty space suggestive of an abortive “lumen” (Figure 2C). This confirms earlier EM studies of 3D cultures revealing a spatial organization in 3D similar to that in the original tumour [20]. Furthermore, tight junctions were identified between adjacent cells (Figure 2D), whereas desmosomes were absent, as reported [21]. This is in agreement with the expression of E-cadherin (CDH1), involved in cell-cell interaction and aggregation, to be increased in 3D compared to 2D culture by RT-PCR and Western blotting (Figure 2E-F). The mRNA expression of the cell adhesion protein E-cadherin increased during the initial phase of spheroid formation and dropped after day 4, indicating low epithelial cell turnover in the spheroid after day 4. In the 2D culture E-cadherin is expressed at later stages when cells make contacts upon reaching confluency. In addition, due to cell-cell contacts over the complete cell surface the E-cadherin protein expression is always higher in the 3D culture compared to the 2D culture where cells only make lateral contacts.

### Altered energy metabolism and lactate accumulation in 3D spheroids

**Figure 3:**
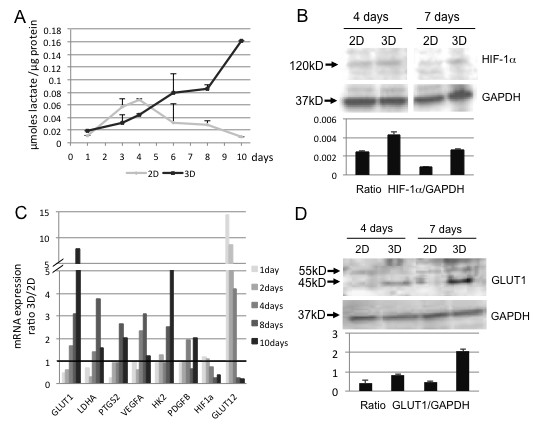
**Metabolical and physiological analyses comparing 2D with 3D culture. [A] **Energy metabolism: lactate accumulation measurements in 3D compared to 2D culture. Data are expressed as ratio μmoles lactate/ μg protein. **[B] **Western immunoblotting showing HIF1a protein expression both in 2D and 3D cultures at day 4 and 7. **[C] **mRNA expression of target genes downstream HIF1a in 2D and 3D cultures. Real Time-PCR data are calculated as expression ratio 3D/2D. A representative experiment out of three is shown. **[D] **Western blotting shows Glut1 protein expression both in 2D and 3D cultures on day 4 and 7. GAPDH is used as loading control.

Growing in 3D induces a different gene expression pattern as compared to 2D [22]. Tumour cell spheroids have many characteristics in common with native cancer, such as gradients for oxygen/hypoxia, nutrients, lactate accumulation, and proliferation and as such they resemble small stroma-embedded cancer cell nests [23]. These different physical and chemical properties modify cell behavior and functions, which together result in a substantially different cellular microenvironment that mimics more closely that of native tissue, e.g. regarding mechanical–chemical signaling in the interstitium and the concentration gradients for nutrition, waste and oxygen [24]. As a principle measure of the cellular energy metabolism we investigated the lactate accumulation in the culture medium at various time points, and results were compared with those from 2D Panc1 cultures. During the first days, lactate is accumulating at similar rates in 2D and 3D cell cultures (Figure 3A). From day 5–6 onward, however, lactate accumulation increased significantly more in 3D than 2D cell culture medium, indicating a metabolic switch to increased glycolysis in 3D. This is called the Warburg effect, ie. the transition of the energy metabolism from oxidative phosphorylation to aerobic glycolysis induced by the lack of oxygen [25], which is even further supported by an increase in the mRNA expression of glucose transporter 1 (GLUT1) and lactate dehydrogenase (LDHA) after the initial sphere forming phase (Figure 3C). Under 2D culture conditions, the lactate content of the medium decreases after 4–5 days of culture without medium change. This indicates, as described earlier [26], that, if nutrients are lacking, growing tumour cells can use the lactate they have produced previously as an ultimate oxidative energy substrate, even in normo-oxygenic conditions. While the use of lactate is impaired by functioning p53, this was absent in all cell lines tested in this study [27]. Recent evidence suggests that lactate itself can induce secretion of hyaluran [28], an ECM constituent expressed by PDAC which binds to CD44 [29,30]. Lactate can also contribute to an increase in VEGF [28], as observed in the 3D model.

**Figure 4:**
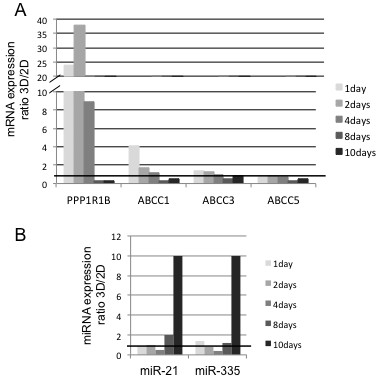
**Analyses of chemoresistance related genes. **[**A**] Time course of mRNA expression in 2D and 3D cultures of drug resistance-involved genes. Real Time-PCR data are calculated as expression ratio 3D/2D. [**B**] Time course expression of drug resistance-relevant miRNAs in 2D and 3D cultures. Real Time-PCR data are calculated as expression ratio 3D/2D. A representative experiment out of three is shown.

The increased lactate in 3D, together with the (mild) hypoxia, could also be thought indicative of cellular stress that had been shown in other tumor cell models to increase MRP1 and P-gp expression, increasing sensitivity for gemcitabine [31]. This seems not to be the case in our 3D model system because we observe a decreasing expression at least for MRP1/ABCC1 (Figure 4A).

As non-vascularized 3D tissue culture may develop hypoxic regions, the expression of HIF-1α and downstream target genes was investigated in both 2D and 3D Panc-1 cultures on days 4 and 7. The total HIF-1α protein level was similar in 2D and 3D cultures at day 4 but was lower at day 7 in 2D culture, whereas it was maintained at the same level in 3D culture (Figure 3B). This indicates that HIF-1α protein stability is higher in cells growing in 3D compared to 2D culture.

In order to corroborate this finding, the expression of genes downstream of HIF-1α, ie. GLUT1, GLUT12, PTGS2, VEGFA, HK2 and PDGFB, was assessed by RT-PCR at various time points (Figure 3C). RNA expression of GLUT1, VEGF and HK2 was found to be higher in 3D compared to 2D culture particularly from day 4 onward, whereas GLUT12 expression was decreased over time. For GLUT1 this was also verified at the protein level (Figure 3D).

### Increased extracellular matrix (ECM) in 3D culture

**Figure 5:**
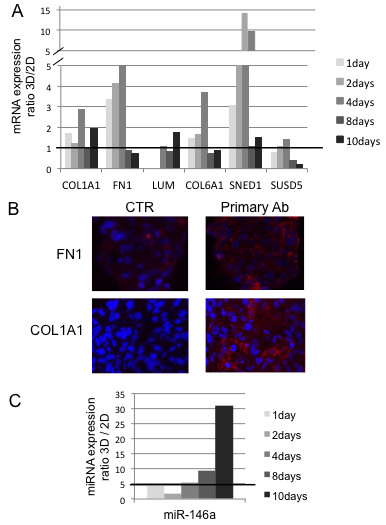
**Analyses of extracellular matrix related genes. **[**A**] mRNA expression of ECM relevant genes in 2D and 3D cultures. Real Time-PCR data are calculated as expression ratio 3D/2D. A representative experiment is shown [**B**] Collagen I and fibronectin I staining of a central section from 7 day spheroids (right). One representative picture is shown. Secondary antibody alone staining is used as negative control (CTR, left). [**C**] ECM-relevant time course of selected miRNA expression in 2D and 3D cultures. Real Time-PCR data are calculated as expression ratio 3D/2D. A representative experiment out of three is shown.

PDAC cells express already endogenous ECM components such as collagen and fibronectin-1 [32] and the respective integrins [33] as a consequence of TGFß1 [34]. We were therefore interested in the effect of a matrix-free 3D culture on the ECM production. We investigated the mRNA expression of relevant genes such as COL1A1 (collagen I), COL6A1 1(collagen VI), FN1 (fibronectin I), LUM (lumican), SNED1 and SUSD5 (sushi domain containing 5) by RT-PCR at various time points in 2D and 3D (Figure 5A). The expression of the ECM genes FN, COL6A1 and COL1A and membrane transporter genes ABCC1/-3/-5 was higher in 3D during the sphere formation (contact making) and compaction phase (Figure 4A and 5A). After day 4 a steady-state-level was reached in 3D with reduced mRNA expression, while the 2D culture grows confluent and the expression of these contact or cell proximity-affected genes went up. The protein expression of collagen I and fibronectin I was confirmed by immunohistochemistry on spheroid sections (Figure 5B). Lumican, a proteoglycan that is frequently expressed in cancer, co-localizes with collagens in many tissues, and has a well-defined biological role in maintaining tissue structural homeostasis [35], was also highly up-regulated in our model and seemed to be expressed also in 2D cultures from day 4 when cells became more confluent. We were also interested in some further molecules bearing distinct capabilities for our 3D model. SNED1 (sushi, nidogen and EGF-like domains 1) a protein identified as a stroma marker [36], was strongly up-regulated, particularly at early time points (day 2–4). Interestingly, it has been identified as a cisplatin-resistance related gene in head and neck squamous carcinoma [37].

Furthermore, we searched for additional modulators of ECM. miRNAs have been described recently as a new class of gene regulators, also in PDAC [38], where some were reported to regulate stromal molecules. mir-146a suppresses invasive cell properties and is under-expressed in Panc-1 cells compared to normal human pancreatic ductal cells [39]. We found a strong up-regulation of mir-146a when Panc-1 cells were grown in 3D (Figure 5C). This may possibly reflect the forced immobilization of cancer cells in the spheroid [40].

### Increased expression of chemoresistance-related genes

Chemoresistance in solid tumors is conveyed by different mechanisms. The classical are based on MDR genes and transporter proteins, all reported to contribute to chemoresistance in PDAC [3,4,41]. We therefore evaluated the mRNA expression of genes involved in drug resistance by RT-PCR in 2D and 3D Panc-1 cultures. The ATP binding cassette ABCC1 was up-regulated during the initial sphere formation period (Figure 4A). Furthermore, expression of miRNAs miR-21 and miR-335 associated with elevated chemoresistance [42-44] was increasing in 2D culture until day 4 and then constantly decreasing until day 10. In contrast, in 3D culture the expression of miR-21 and miR-335 peaked later on day 8, decreasing slightly thereafter, resulting in higher expression (Figure 4B). There are other molecules described more recently. PPP1R1B (protein phosphatase1, regulatory subunit1B) formerly called DARPP-32, is a bifunctional signal transduction molecule acting both as kinase and phosphatase inhibitor, that has been detected in several solid tumours including some carcinomas of the GI tract. The truncated form, t-DARPP-32, has been demonstrated to confer drug resistance, e.g. against trastuzumab in breast cancer via the AKT pathway, or against gefitinib in gastric cancer via EGFR/ERBB3 [45] and by reducing drug-related apoptosis via CREB/PKA [46]. T-DARPP is also responsible for the nuclear translocation of ß-catenin [47]. We found it highly upregulated in the 3D culture system. SNED1, as described above, conveys drug resistance against platinum [37]. Finally, PDAC cells become more resistant to drugs if cultivated on fibronectin or collagen I, both found upregulated (see above), indicating a role for these ECM proteins in protecting cells from chemotherapy [48,49]. Due to increased extracellular matrix *in vitro* 3D systems provide mechanical properties that act as a barrier to drug diffusion [49,50]. Collagen I, for example, a major component of ECM, is expressed at a higher level in 3D than in 2D breast cancer cell cultures [9]. This observation is of particular interest, as collagen I is involved in gemcitabine resistance in pancreatic cancer [51]. Fibronectin-1, which mediates cell and tissue cohesion, is also up-regulated in pancreatic and other cancers [52-54].

In other tumor cell models, cellular stress caused MRP1 and P-gp overexpression leading to increased Gemcitabine sensitivity, which could be abolished by blocking these efflux pumps with verapramil [31].

### Increased chemoresistance in 3D culture

**Figure 6:**
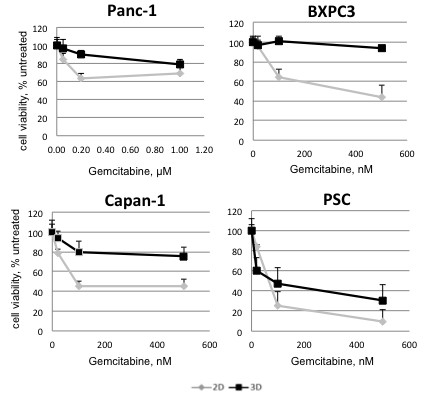
**Increased chemoresistance against gemcitabine in 3D culture. **Cell viability after Gemcitabine treatment of different PDAC cell lines grown in 2D and 3D culture. A Pancreatic Stellate Cell line (PSC) is included as non-transformed control cell line. Data are plotted as percentage of untreated control cells. A representative experiment out of three is shown.

**Figure 7:**
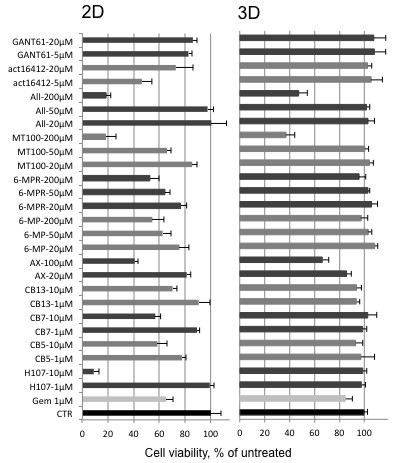
**Comparison of chemoresistance between 2D and 3D culture using multiple cytotoxic compounds. **Histogram summarizing the results from viability assays performed on 2D and 3D Panc-1 cell cultures. Different drugs were used at the indicated concentrations. Data are plotted as percentage of the respective untreated control (CTR) and each drug was tested three times in octuplets. Gem: gemcitabine. All: allicin. AX: AXP-107-11.

Beside the molecules resulting in increased chemoresistance, we were also interested whether we could identify novel substances that would be capable of acting in 3D. A difference in sensitivity to Gemcitabine, the standard for pancreatic cancer treatment, between 2D and 3D culture systems, as described previously [15], was verified in this study as a control: in 2D cultures Gemcitabine reduced cell viability of BXPC3 and Capan-1 to 40-60%, whereas Panc-1 cells were rather resistant to the treatment, and higher Gemcitabine concentrations were required to affect cell viability (around 95% viability left at 100 nM concentration) (Figure 6). As expected, PSC cells included as a non-transformed control cell line were the most sensitive to treatment both in 2D and 3D cultures (20% viability with 100 nM GEM). A panel of drugs with different targets (see Table 1 and for compound details Additional file 2: S2) was tested at two or three concentration levels on both 2D and 3D cultures (Figure 7). Many of the compounds tested, including the microtubule inhibitors CB5 and CB7, the anti-metabolites MT100, allicin, and the flavonoid AXP reduced cell viability to 20-60% at the highest concentration in 2D culture. The effect of the same compounds on the 3D culture was much lower and only a few reduced cell viability maximal to 65% (AXP) and approximately 40% (allicin and MT100)(Figure 7 and Table 1).

The mode of action and molecular mechanisms of these two compounds are subject of further studies.

Testing drugs in a 3D culture model raises the issue of drug penetration, which may be impaired by structural features of the three dimensional culture, including the size of the spheroids [24]. Drug penetration into the spheroid is also determined by diffusion through the ECM. The specific interactions between cancer cells and their microenvironment, both cell-cell and cell-matrix adhesion, are amongst the factors that determine the effect of chemotherapy [55], and are likely to vary from one cell type to another. PDAC cells express already endogenous ECM components such as collagen and fibronectin-1 [32]. Higher drug resistance was shown in PDAC cells grown on fibronectin-1 or collagen coated culture dishes [49]. In our study the acquisition of elevated drug resistance of cancer cells in the 3D culture model may be explained by the increased endogenous ECM protein expression within the microenvironment of the spheroids, thus supporting the proposed cell adhesion-mediated drug resistance (CAM-DR), and upregulation of other, more recently identified molecules described above, e.g. ABC transporters, PPP1R1B, SNED1. However, since we have only tested a limited number of transporters, we can not exclude that other transporters such as P-glycoprotein may play a role, as described in other solid tumor cells in vitro [31].

### 3D culture of pancreatic tumour cells from KRAS mouse model

**Figure 8:**
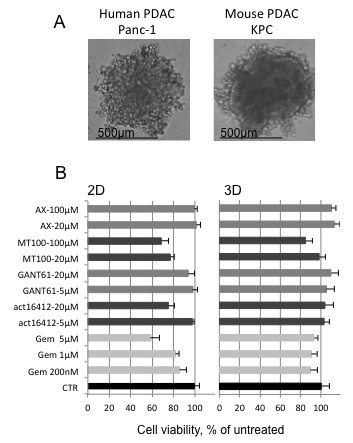
**Spheroids from mouse pancreatic cancer. [A] **PDAC cells from a mouse bearing mutated Kras and Trp53 in the pancreas (KPC cells) are compared to the human PDAC Panc-1 cell line in their ability to form spheroids when grown under 3D culture conditions. **[B] **Drug assay performed on KPC cells. Different compounds are used at the indicated concentrations and cell viability of 2D versus 3D culture is compared. Data are plotted as percentage of the respective untreated control (CTR). A representative experiment out of three is shown.

Having gone through numerous passages, established cancer cell lines bear the risk of differing to a more or less significant extent from their original parent cell line. To validate and confirm the above findings, experiments were also performed on a cell line that was freshly established from the current state-of-the-art pancreatic cancer mouse model with Kras and p53 mutations in the pancreas (Kras^LSL-G12D/+^;Trp53^LSL-R172H/+^;p48-Cre, KPC) [14]. These cells, used at low passage numbers, were able to form spheroids under the same conditions as Panc-1, and after 4 days the spheroid size reached the same range (500 μM) as that of Panc-1 spheroids (Figure 8). Various drugs were tested on KPC cells in both 2D and 3D culture, and cell viability was measured. A higher drug resistance observed in cells grown in 3D compared to 2D conditions validated and extended the findings from human PDAC cell lines (Figure 8).

## Conclusions

For decades, conventional two-dimensional (2D) cell culture has been the cornerstone of screening of novel drugs for pancreatic cancer as much as for other solid tumours [56]. It represents a convenient and high-throughput but rather artificial method of growing cells. Nonetheless, the predictive value was satisfactory, especially in non-solid malignancies.

As cellular response to drugs is profoundly affected by microenvironmental factors, the use of a 3D-culture seems more appropriate for drug testing. This applies in particular to tumours such as PDAC, which are chemoresistant in most patients, despite a good response in (2D) tissue culture and xenograft models [57]. The newly described genetically engineered mouse models, namely the KP and KPC mouse, better recapitulate the impact that inflammatory and stromal cells have in the pathogenesis of PDAC [14].

Our results confirm the previously described increased chemoresistance in 3D; we further demonstrate a more matrix-rich phenotype in 3D culture that may be advantageous for drug testing as it simulates more closely the *in vivo* situation: in 3D culture the microenvironment acquires new features with altered ECM composition, which has a major role in protecting the cells from drug activity [10,58]. Expression of several key matrix proteins and miRNAs related to stromal development is increased, as is glycolysis. These changes mirror features that are characteristic of PDAC, i.e. a high content in ECM components [32] and growth factors such as PDGFB and VEGF, which are responsible for tumour progression.

**Figure 9:**
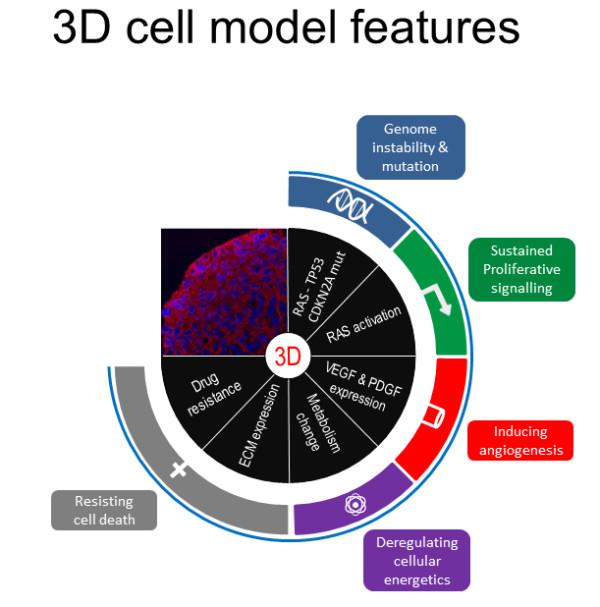
**3D spheroid model. **Representation of the characteristics of the 3D spheroid model in relation to the holistic hallmarks of cancer according to Hanahan and Weinberg [59] observed in the reductionist pancreatic cancer monospheroid model.

In summary, up-regulation of several key ECM components in conjunction with a differentiated deregulation of selective miRNAs and several other novel molecules, eg SNED1 and DARP32, in 3D PDAC cell culture is indicative of a more matrix-rich and at the same time more chemoresistant phenotype. The observed poorer response to a selection of drugs, including several new substances, in 3D compared to 2D cell culture corroborates this notion. Our data support two of the three mechanisms that are proposed to underlie chemoresistance according to the novel hypothesis of cell adhesion mediated drug-resistance (CAM-DR) [10]: spheroid formation and matrix/fibronectin production (the third mechanism being related to the stroma). The observations in human PDAC cell cultures were mirrored by identical results using primary PDAC cells from the KPC mouse model, thereby underscoring the universality of the phenomenon. Taken together, the switch from 2D to 3D growth affects several “hallmarks of cancer” and leads to a more aggressive cancer phenotype [59] (Figure 9).

In addition to elucidating the mechanisms of chemoresistance and the role of CAM-DR in PDAC [4], the 3D model characterized in this study may serve as a high-throughput screening platform for chemotherapeutic drug testing that provides a more reliable prediction of the response to treatment of patients with pancreatic cancer.

## Competing interests

There are no competing interests for publication.

## Authors’ contributions

ML and RH together with PL and XJ designed the experiments. PL, XJ, JE and AW conducted the experimental work. CV reviewed the histology. MRW, SR and RT contributed drug substances. All were involved in the discussion of the results. ML, RH, PL and XJ mainly wrote the manuscript. All authors contributed and approved the final version.

## Pre-publication history

The pre-publication history for this paper can be accessed here:

http://www.biomedcentral.com/1471-2407/13/95/prepub

## Supplementary Material

Additional file 1 Real Time PCR. Click here for file

Additional file 2 Experimental drugs used in 2D and 3D cultures. Click here for file

Additional file 3 Acid Phosphatase Assay. Click here for file
