# Supplementary material for: 3D pancreatic carcinoma spheroids induce a matrix-rich, chemoresistant phenotype offering a better model for drug testing
Source: BMC Cancer. 2013 Feb 27;13:95. doi: 10.1186/1471-2407-13-95 (PMC3617005; doi:10.1186/1471-2407-13-95)
Supplement: Additional file 1 — Real Time PCR. [file 1471-2407-13-95-S1.doc]

Additional File 1

**Real Time PCR**

Each PCR reaction mixture of 25µl, contained 4 µl of template cDNA (diluted 1:20), 12.5 μl of SYBR-Green reagent, 1µl each specific primer (final concentration 0,4µM) and 6.5μl sterile water. Thermal cycle protocols were performed for 40 cycles of 10 s at 95◦C, 30 s at 60◦C. All PCR reactions were carried out in triplicates using a C1000 Thermal Cycler CFX96 Real time System (Biorad). To confirm amplification specificity, PCR products were subjected to a melting curve analysis. Primers for RT-PCR were selected at PCR primer databases (PrimerDepot and Roche Universal Probe Library: [**http://primerdepot.nci.nih.gov/**](http://primerdepot.nci.nih.gov/)) and purchased from Invitrogen (sequences in Table 1).

Table 1

List of primers used for PCR for the respective genes.

| **Gene** | **amplicon** | **Forward** | **Reverse** |
| --- | --- | --- | --- |
| ABCC1 | 92 | TGGGCAGGGATTCTCTTTTA | TCATGCTCACTTTCTGGCTG |
| ABCC3 | 116 | AGCTCGGCTCCAAGTTCTG | GACCCACAGGTAGATGCAGG |
| ABCC5 | 103 | CTGCTGTTTCCAAGGCATCT | GTGAGGGAGAGAACCAGCAC |
| CDH1 | 93 | GACCGGTGCAATCTTCAAA | TTGACGCCGAGAGCTACAC |
| COL1A1 | 91 | CACACGTCTCGGTCATGGTA | AAGAGGAAGGCCAAGTCGAG |
| COL6A1 | 61 | CCTGGAGGGCTACAAGGAA | GTGCTTGGCCTCGTTCAC |
| FN1 | 76 | GGTGGAATAGAGCTCCCAGG | GCAGCCTGCATCTGAGTACA |
| HIF1a | 149 | GTGGAAGTGGCAACTGATGA | ATTCACCATGGAGGGCG |
| HK2 | 77 | GCTGCAGAGATGCTCTTTTTC | TTCGCTTGCCCCATTATC |
| LUM | 130 | CCCCAGGATCTTGCAGAAG | GGTTGAGCTGGATCTGTCCT |
| PDGFB | 105 | AATGGTCACCCGAGTTTGG | CTGGCATGCAAGTGTGAGAC |
| PPP1R1B | 113 | CACACCACCTTCGCTGAAA | GAAGCTCCCCCAGCTCAT |
| PTGS2 | 103 | CCGGGTACAATCGCACTTAT | GGCGCTCAGCCATACAG |
| SLC2A1 | 344 | AGCCTGTGTATGCCACCATT | GCCCACAATGAAATTTGAGG |
| SLC2A12 | 61 | TGCTGCTTTTTCAATTGGTCT | AGGAAAGATCTCGCTGAGCA |
| SNED1 | 96 | GCTCGAGAACATGGAGGAAG | CAGTTGCCAGGGACGTTT |
| SUSD5 | 75 | GGAGTGGTACGGCCTGGT | GGGAAGTTATCTTCATAGTCAATGTG |
| VEGFA | 119 | GCAGTAGCTGCGCTGATAGA | CCTTGCTGCTCTACCTCCAC |

For miRNAs detection, universal qPCR primer, miR-21(miR#1315) and mi-R146a (miR#1409) were from Invitrogen, whereas U6snRNA (#600750) and miR-29a (#600719) were from Stratagen Agilent Technologies (San Diego, CA, USA). In order to avoid potential interference of the cell culture conditions with the expression of typically used cytoskeletal markers, the ribosomal protein RPLP13 was used as housekeeping gene (1). For microRNAs the small nuclear RNA U6 was used to normalize the microRNA expression levels. The analysed RT-PCR data were always normalized against the sample 2D culture at day 1 and expression ratios 3D/2D were calculated. Expression ratios above one or below one indicate increased or decreased expression in the 3D compared to the 2D culture, respectively. Expression ratio close to one indicates no expression difference.

**REFERENCES**

1. Jesnowski R, Backhaus C, Ringel J, et al. Ribosomal highly basic 23-kDa protein as a reliable standard for gene expression analysis. Pancreatology. [Research Support, Non-U.S. Gov't]. 2002;2(4):421-4.
