# Supplementary material for: 3D pancreatic carcinoma spheroids induce a matrix-rich, chemoresistant phenotype offering a better model for drug testing
Source: BMC Cancer. 2013 Feb 27;13:95. doi: 10.1186/1471-2407-13-95 (PMC3617005; doi:10.1186/1471-2407-13-95)
Supplement: Additional file 2 — Experimental drugs used in 2D and 3D cultures. [file 1471-2407-13-95-S2.doc]

Additional File 2

**Experimental drugs used in 2D and 3D cultures.**

The following drugs were used: gemcitabine (GEM = Gemzar, Eli Lilly) stocks (40 mg/ml solution) were stored in aliquots at –20°C and single aliquots were utilized for the drug treatment. We used the microtubule inhibitors CB5 (Chembridge code 5248881), CB7 (Chembridge code 5276937), CB13 (Chembridge code 5350849) (1, 2), the sHH inhibitors Act-16412 (Actar AB, Stockholm, Sweden) and GANT61 (3), the genisteine analogue AXP-107-11 (Axcentua AB, Stockholm, Sweden) as well as allicin (4) and the MT100 (S-allylthio-6-mercaptopurine) (5). Both Allicin and MT-100 were provided by Talia Miron (Allylea, Israel). Experimental drugs were used at the indicated concentrations.

**REFERENCES**

1. D'Arcy P, Brnjic S, Olofsson MH, et al. Inhibition of proteasome deubiquitinating activity as a new cancer therapy. Nat Med. 2011;17(12):1636-40.

2. Fayad W, Rickardson L, Haglund C, et al. Identification of agents that induce apoptosis of multicellular tumour spheroids: enrichment for mitotic inhibitors with hydrophobic properties. Chem Biol Drug Des. [Research Support, Non-U.S. Gov't]. 2011 Oct;78(4):547-57.

3. Lauth M, Bergstrom A, Shimokawa T, et al. Inhibition of GLI-mediated transcription and tumor cell growth by small-molecule antagonists. Proceedings of the National Academy of Sciences of the United States of America. [Research Support, Non-U.S. Gov't]. 2007 May 15;104(20):8455-60.

4. Bat-Chen W, Golan T, Peri I, et al. Allicin purified from fresh garlic cloves induces apoptosis in colon cancer cells via Nrf2. Nutr Cancer. [Research Support, Non-U.S. Gov't]. 2010;62(7):947-57.

5. Miron T, Arditti F, Konstantinovski L, et al. Novel derivatives of 6-mercaptopurine: synthesis, characterization and antiproliferative activities of S-allylthio-mercaptopurines. Eur J Med Chem. 2009 Feb;44(2):541-50.
