# Supplementary material for: 3D pancreatic carcinoma spheroids induce a matrix-rich, chemoresistant phenotype offering a better model for drug testing
Source: BMC Cancer. 2013 Feb 27;13:95. doi: 10.1186/1471-2407-13-95 (PMC3617005; doi:10.1186/1471-2407-13-95)
Supplement: Additional file 3 — Acid Phosphatase Assay. [file 1471-2407-13-95-S3.doc]

Additional File 3

**Acid Phosphatase Assay**

The APH assay, which measures the activity of acidic phosphatase in the cells, was optimized for 3D cultures as follows. Only 70µl of medium were removed in order not to suck up the spheres. Then 60µl of PBS was added per well and 100µl of APH buffer (0.1M sodium acetate pH 5.2 and 0.1% triton X-100) containing the substrate (2mg/ml *p*-nitrophenyl phosphate, final pH 4.8 from Thermo Scientific). After 2 hours incubation for the 2D culture and 5 hours for the 3D culture, 10µl of 1M NaOH were added to stop the APH activity. Absorbance at 405nm was measured within 10 minutes using a FluoStar OPTIMA plate reader (BMG Labtech). Viability rate was calculated as per cent of CTR untreated cells. All data are expressed as the mean ± SD of at least 8 replicates. All experiments were performed at least three times.
